# Associations between PM_2.5_ and Heart Rate Variability Are Modified by Particle Composition and Beta-Blocker Use in Patients with Coronary Heart Disease

**DOI:** 10.1289/ehp.11062

**Published:** 2008-08-25

**Authors:** Jeroen J. de Hartog, Timo Lanki, Kirsi L. Timonen, Gerard Hoek, Nicole A.H. Janssen, Angela Ibald-Mulli, Annette Peters, Joachim Heinrich, Tuula H. Tarkiainen, Rene van Grieken, Joop H. van Wijnen, Bert Brunekreef, Juha Pekkanen

**Affiliations:** 1 Institute for Risk Assessment Sciences, Utrecht University, Utrecht, the Netherlands; 2 Environmental Epidemiology Unit, National Public Health Institute, Kuopio, Finland; 3 Department of Clinical and Nuclear Medicine, Kuopio University and Kuopio University Hospital, Kuopio, Finland; 4 National Institute for Public Health and Environment, Bilthoven, the Netherlands; 5 Institute of Epidemiology, Helmholtz Zentrum München – German Research Center for Environmental Health, Neuherberg, Germany; 6 Department of Clinical Physiology and Nuclear Medicine, Mikkeli Central Hospital, Etelä-Savo Hospital District, Mikkeli, Finland; 7 Department of Chemistry, University of Antwerp, Antwerp, Belgium; 8 Department of Environmental Medicine, Municipal Health Service Amsterdam, Amsterdam, the Netherlands; 9 Julius Center for Health Sciences and Primary Care, University Medical Center Utrecht, Utrecht, the Netherlands; 10 School of Public Health and Clinical Nutrition, University of Kuopio, Kuopio, Finland

**Keywords:** absorbance, air pollution, cardiovascular health, elements of PM_2.5_, heart rate variability, medication, PM_2.5_, source-specific particulate matter

## Abstract

**Background:**

It has been hypothesized that ambient particulate air pollution is able to modify the autonomic nervous control of the heart, measured as heart rate variability (HRV). Previously we reported heterogeneous associations between particulate matter with aerodynamic diameter < 2.5 μm (PM_2.5_) and HRV across three study centers.

**Objectives:**

We evaluated whether exposure misclassification, effect modification by medication, or differences in particle composition could explain the inconsistencies.

**Methods:**

Subjects with coronary heart disease visited clinics biweekly in Amsterdam, the Netherlands; Erfurt, Germany; and Helsinki, Finland for 6–8 months. The standard deviation (SD) of NN intervals on an electrocardiogram (ECG; SDNN) and high frequency (HF) power of HRV was measured with ambulatory ECG during paced breathing. Outdoor levels of PM_2.5_ were measured at a central site. In Amsterdam and Helsinki, indoor and personal PM_2.5_ were measured during the 24 hr preceding the clinic visit. PM_2.5_ was apportioned between sources using principal component analyses. We analyzed associations of indoor/personal PM_2.5_, elements of PM_2.5_, and source-specific PM_2.5_ with HRV using linear regression.

**Results:**

Indoor and personal PM_2.5_ were not associated with HRV. Increased outdoor PM_2.5_ was associated with decreased SDNN and HF at lags of 2 and 3 days only among persons not using beta-blocker medication. Traffic-related PM_2.5_ was associated with decreased SDNN, and long-range transported PM_2.5_ with decreased SDNN and HF, most strongly among persons not using beta blockers. Indicators for PM_2.5_ from traffic and long-range transport were also associated with decreased HRV.

**Conclusions:**

Our results suggest that differences in the composition of particles, beta-blocker use, and obesity of study subjects may explain some inconsistencies among previous studies on HRV.

Increased cardiovascular mortality and morbidity have been reported in association with increases in daily ambient levels of particulate matter (PM) in epidemiologic studies (Analitis et al. 2006; Le Tertre et al. 2002; Samet et al. 2000). However, it is not known which constituents of particles are responsible for the effects associated with particle mass. The source of particles defines their composition. Recent epidemiologic studies suggest that particles from combustion sources are especially harmful (Laden et al. 2000; Lanki et al. 2006). Transition metals and organic carbon compounds were shown to be toxic in a toxicologic study (Pagan et al. 2003). These can be found in abundance in combustion particles.

The relative importance of different pathways from particle exposure to effects on the cardiovascular system is not clear, but exposure to particles has been associated both with increased systemic inflammation and changes in autonomic nervous control of the heart (Brook et al. 2004). The latter is most often measured indirectly as heart rate variability (HRV) (Task Force 1996). A decreased overall HRV has proven to be a strong independent predictor of cardiac mortality in subjects with existing cardiovascular disease (La Rovere et al. 1998; Nolan et al. 1998). Several studies have shown decreased indices of HRV on days with increased outdoor levels of respirable particles [aerodynamic diameter < 10 μm (PM_10_)] (Liao et al. 2004; Lipsett et al. 2006) and fine particles [< 2.5 μm (PM_2.5_)] (Holguín et al. 2003; Schwartz et al. 2005).

In the Exposure and Risk Assessment for Fine and Ultrafine Particles in Ambient Air (ULTRA) study, levels of outdoor air pollution were monitored for 6–8 months in 1998–1999 in three European cities. At the same time, panels of patients with coronary heart disease were followed up with measurements of HRV. We previously reported that the levels of ultrafine particles (PM < 0.1 μm) were associated with changes in the balance between sympathetic and vagal nervous input to the heart (Timonen et al. 2006). However, PM_2.5_ in particular showed different associations with HRV in the different study centers. In Helsinki, Finland, elevated concentrations of PM_2.5_ were associated with decreased high frequency (HF) and increased low frequency (LF)/HF ratio, whereas the opposite was true in Erfurt, Germany. No such associations were observed in Amsterdam, the Netherlands.

In the present article, we evaluate whether exposure misclassification, effect modification by medication, or variable particle composition could explain these inconsistencies. Personal and indoor PM_2.5_ were measured in Amsterdam and Helsinki to obtain more accurate estimates of exposure. Possible effect modification by beta-blocker (β-adrenergic antagonist) medication or obesity was evaluated in light of the limited number of earlier studies (Chen et al. 2007; Park et al. 2005; Wheeler et al. 2006). For comparison, we also tested other medication possibly modifying the effect of particulate air pollution on HRV.

Finally, we linked source-specific PM_2.5_ with HRV to evaluate the importance of particle composition for cardiovascular effects of PM.

## Methods

Heart rate variability was measured at biweekly clinic visits in panels of elderly subjects with coronary heart disease in Amsterdam, Erfurt, and Helsinki in 1998–1999. In Amsterdam, 37 panelists were followed for 8 months, and in Erfurt and Helsinki, 47 panelists were followed for 6 months. The visits of every subject were always scheduled for the same weekday and for the same time. The medication of the subjects was not changed for the clinic visits. Outdoor levels of PM_2.5_ were measured concurrently with the visits at one central site in each city. In Helsinki and Amsterdam, indoor and personal measurements of PM_2.5_ were also performed during the 24 hr preceding the clinic visit. All measurements in the study were performed according to standard operating procedures (Brunekreef et al. 2005; Pekkanen et al. 2000).

The main inclusion criteria for the study were a self-report of a physician-diagnosed coronary heart disease, being a nonsmoker, and age = 50 years. Ethical committees in each study center approved the study protocol. A written informed consent was obtained from all subjects.

At the clinic visits, HRV was recorded with an ambulatory electrocardiogram (ECG) recorder (Medilog MR 63 recorder; Oxford Instruments, Abington, UK) using a standardized protocol (Timonen et al. 2006). Breathing frequency strongly affects HRV, and for that reason, HRV recorded during a 5-min period of paced breathing in supine position (frequency 0.2 Hz; 2.5-sec inhalation and 2.5-sec exhalation) has been used for the analyses. Two-channel ambulatory ECG recordings were performed with analog ambulatory ECG recorders (Medilog MR 63 recorder; Oxford Instruments) using standard electrode position for leads V1 and V5. The recordings were analyzed with ambulatory ECG analysis software (Exel Medilog II V7.5 system; Oxford Instruments). The recordings were digitized with a sampling rate of 128 Hz. The software used an interpolation algorithm to refine the R wave fiducial point and to improve the resolution in R-peak detection. Details of the analyses have been published previously (Tarkiainen et al. 2005; Timonen et al. 2006).

We were mainly interested in explaining the heterogeneous results in the main end points [the SD of NN intervals (SDNN), HF, and LF/HF ratio] of a previous ULTRA paper (Timonen et al. 2006). Therefore, we used two common indices of HRV in the present analyses: SDNN, which is a time-domain measure of overall HRV, and HF power (0.15–0.4 Hz) of HRV, which is a frequency domain measure believed to reflect mainly the vagal (parasympathetic) part of the autonomic nervous input to the heart. HF is highly correlated with r-MSSD, a commonly used time-domain variable (Task Force 1996). The LF/HF ratio was left out of the paper, because the interpretation and physiological basis are more controversial.

Information on physician-administrated daily medication was collected at baseline visit. Medication categories tested for effect modification were beta-blockers, calcium (Ca^2+^) channel blockers, statins, angiotensin-converting enzyme (ACE) inhibitors, angiotensin receptor blockers, and acetylsalicylic acid (ASA). Antiarrhythmic medication was not included in the analyses because of limited use among study participants (7%).

Harvard impactors (BGI, Inc., Waltham, MA, USA) were used to collect filter samples of outdoor PM_2.5_; GK2.05 cyclones and battery-operated AFC400S pumps (BGI, Inc.) were used for the collection of personal PM_2.5_ samples. The filters were weighted to determine mass of PM_2.5_, and reflectance was measured with a reflectometer (Model 43, Diffusion Systems Ltd., London, UK). The reflectance was transformed into absorbance [absorption coefficient (ABS)], which is an indicator for elemental carbon. Finally, elemental composition of the samples was determined using energy-dispersive X-ray fluorescence spectrometry. All methods have been described in detail in previous papers (Brunekreef et al. 2005; de Hartog et al. 2005; Janssen et al. 2000, 2005).

We used principal-component analysis and multivariate linear regression to apportion PM_2.5_ mass to different sources (Vallius et al. 2005), thereby obtaining estimates of daily source-specific PM_2.5_ concentrations. Besides components of PM_2.5_ (elemental concentrations and absorbance), daily data on ultrafine (diameter < 0.1 μm) and accumulation mode particles (0.1–1.0 μm), nitrogen dioxide, and sulfur dioxide were used to identify sources.

We identified four to six main source categories in each city: local traffic (with contribution from other local combustion sources), long-range transported (secondary) air pollution, industry, crustal, oil combustion, and salt (Vallius et al. 2005).

We analyzed data using the SAS statistical package and mixed models (PROC MIXED) (SAS Institute Inc., Cary, NC, USA) taking into account repeated observations and assuming constant correlation between observations within a subject. A basic model was first built without including particulate air pollution in the model. Criteria for building the basic model were Akaike’s information criterion and covariate-response plots. The same basic models as in the previous paper have been used (Timonen et al. 2006). Lag 0 was defined as the 24-hr period from noon of the day of the clinic visit to noon of the previous day, lag 1 was the previous 24-hr period, and so on. In Amsterdam, the model included linear terms for time trend, temperature (lag 2), relative humidity (lag 3), and barometric pressure. In Erfurt, the model included linear terms for time trend, relative humidity (lag 3), and barometric pressure (lag 2). Temperature (lag 3) was modeled with linear, squared, and cubic terms. The basic model for Helsinki included linear terms for time trend, relative humidity (lag 1), and barometric pressure (lag 1). Temperature (lag 3) was modeled with linear and squared terms. In all cities, the model included weekday as a categorical variable. Results were insensitive to alternative model specifications.

For comparison of the effects of outdoor, indoor, and personal PM_2.5_ on HRV, only the days with all three types of measurements were included in the analyses. We analzed associations of source-specific PM_2.5_ with HRV using multipollutant models that included at the same time all identified sources and the fraction. Multipollutant unidentified PM_2.5_ models were not used for elements of PM_2.5_ because of high intercorrelations. We analyzed data only for the elements that are either indicators for the PM_2.5_ sources or that have been found harmful in toxicologic studies. The indicators were chosen based on the elemental profiles of sources (Vallius et al. 2005): absorbance for local traffic; sulfur for long-range transported particles; vanadium for oil combustion (not used for Erfurt because oil source was not identified there, and > 50% of concentrations were below detection limit); Ca for soil particles; and chloride for salt particles (not in Erfurt). Elements considered because of potential toxicity were the transition metals copper, iron, and zinc.

HF was log-transformed for the analyses, and the effect of particulate air pollution on the end point was estimated as percent change: [e^(β × IQR)^−1] × 100%, where β is the estimated regression coefficient and IQR is the interquartile range. Effect estimates for the elements are presented for increases that are close to study mean interquartile ranges (IQRs)—the differences between the 25th and 75th percentiles of the exposure distributions. Pooled effect estimates were calculated as a weighted average of the center-specific estimates using the inverse of center-specific variances as weights. The heterogeneity of effect estimates between centers was tested with a chi-square test (Normand 1999).

Effect of extreme source-specific PM_2.5_ values on the results was evaluated by excluding at each lag the concentrations that were more than three times the IQR.

## Results

There were 424 clinical visits in Amsterdam, 491 in Erfurt, and 519 in Helsinki. Although special care was given to attachment of the electrodes, some ECG recordings were unsuccessful. There were 366 successful recordings (from 33 patients) in Amsterdam, 432 (44) in Erfurt, and 468 (45) in Helsinki.

In Helsinki, the proportion of males and females was almost equal, but in Amsterdam the panel contained mostly males and in Erfurt almost exclusively males (Table 1). Obesity was common in Helsinki, where one-third of the study subjects were obese (17 persons). There were clearly fewer obese persons in Amsterdam and Erfurt (7 in both). The most commonly used medication was ASA. About two-thirds of the study subjects in Erfurt and Helsinki had daily beta-blocker medication, whereas only about one-third of the subjects were on medication in Amsterdam. Except for SDNN in Amsterdam, HRV indices were lower among beta-blocker users than among nonusers.

Outdoor levels of PM_2.5_ were lower in Helsinki than in Amsterdam and Erfurt (Table 2). In Helsinki, about half of PM_2.5_ was of secondary origin, that is, could be considered long-range transported; in Amsterdam and Erfurt, this was about one-third. Industrial sources of PM_2.5_ were not identified in Helsinki (Vallius et al. 2005). Oil combustion and salt as sources of PM_2.5_ were not identified in Erfurt, and the indicator elements for these sources have not been included.

PM_2.5_ (total) correlated most strongly with long-range transported PM_2.5_, and the correlation with S, the indicator element for this source, was even higher (Table 3). Transition metals Zn, Fe, and Cu correlated highly with absorbance, with the correlation highest for Cu in Amsterdam (*r* = 0.83) and lowest for Fe in Helsinki (*r* = 0.49) (data not shown).

The medians of individual averages (number of measurements) of outdoor, indoor, and personal PM_2.5_ in Amsterdam were 21.0 (417), 14.9 (411), and 15.3 (338) μg/m^3^, respectively. The respective PM_2.5_ levels in Helsinki were 12.0 (478), 10.2 (503), and 10.0 (336) μg/m^3^ (Janssen et al. 2000).

**Figure 1:**
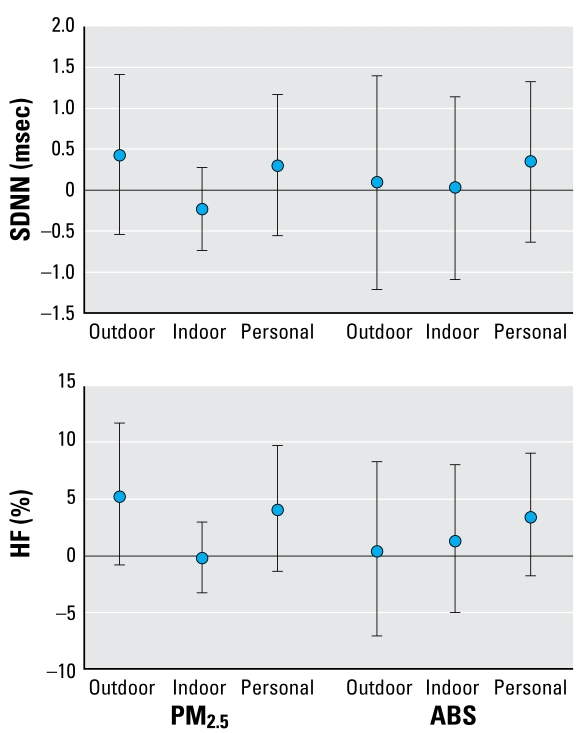
Pooled effect estimates (95% CIs) for two study panels (Amsterdam and Helsinki) for the association outdoor, indoor, and personal PM_2.5_at 0-day lag with HRV (SDNN and HF). Effect estimates are calculated for an increase of 10 μg/m^3^ for PM_2.5_ and 1 m^−1^ × 10^−5^ for absorbance.

Outdoor, indoor, and personal PM_2.5_ were not associated with SDNN at lag 0 (Figure 1). Indoor and personal PM_2.5_ measurements were not available at lags 1, 2, or 3. There was a suggestive positive association of outdoor and personal PM_2.5_ with HF.

**Figure 2:**
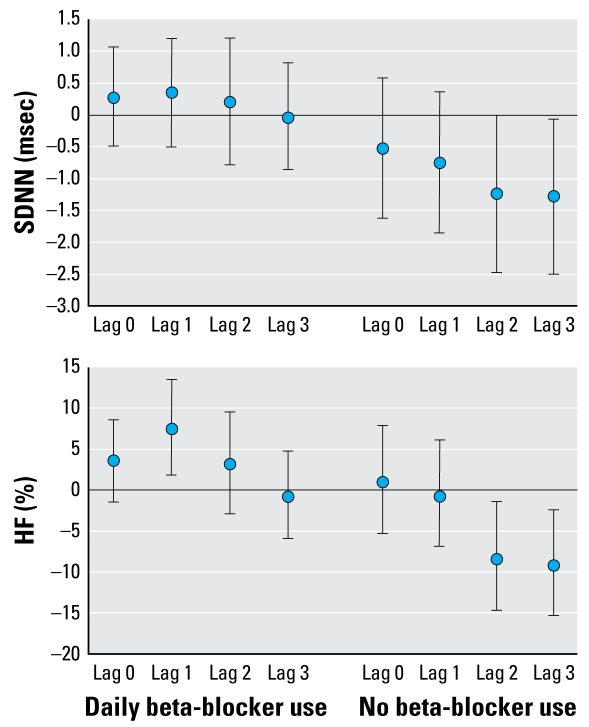
Pooled effect estimates (95% CIs) for three study panels for the association of outdoor PM_2.5_ with HRV (SDNN and HF) stratified by beta-blocker use. Effect estimates are calculated for an increase of 10 μg/m^3^ for PM_2.5_

Among study subjects not on daily beta-blocker medication, increased concentrations of PM_2.5_ were associated with decreased SDNN and HF, especially at longer lags (Figure 2). For this group the city-specific estimates were homogeneous. There was a positive association at single (1-day) lag between PM_2.5_ and HF among subjects who were on medication.

There was no consistent modification of the effects of PM sources by medication other than beta-blockers (results not shown). Those not using ACE inhibitors or angiotensin receptor blockers had more clearly decreased HF in association with long-range transported PM than all subjects [at lag 2: −1.25; 95% confidence interval (CI), −2.09 to −0.41; at lag 3: −1.1; 95% CI, −2.04 to −0.26], but same kind of modifying effect was not observed for other sources or SDNN. On the other hand, those not using statins had decreased HF in association with PM_2.5_ at a 3-day lag (−6.45; 95% CI, −11.63 to −0.96), but no modifying effect of statins was observed for source-specific PM_2.5_ or SDNN.

Obesity was not associated with beta-blocker use: 60.0% of obese and 60.4% of non-obese persons used beta-blockers. However, obesity itself seemed to modify the effects of PM_2.5._ At a 3-day lag, PM _2.5_ was associated with SDNN (−1.99; 95% CI, −3.69 to −0.30) and HF (−12.50; 95% CI, −20.1 to −4.24) among obese persons, whereas such an effect was not observed among all subjects. Effects of long-range transported PM_2.5_ were similarly modified by obesity (results not shown), obviously because of substantial correlation between PM_2.5_ and long-range transported PM_2.5_. However, no such effect modification was observed for PM_2.5_ from traffic or other sources of PM_2.5_.

Increases in PM_2.5_ originating from local traffic were consistently associated with decreased SDNN, somewhat more strongly among study subjects not using beta-blockers than in the whole study panel (Table 4). Long-range transported PM_2.5_ was associated with decreased SDNN and HF at lags 2 and 3 among persons not having daily beta-blocker medication. Among all subjects, there was heterogeneity in the effect estimate for long-range transported PM at a 2-day lag for HF because of negative estimates in Amsterdam (−0.91; 95% CI, −2.02 to 0.22) and Helsinki (−1.92; 95% CI, −3.26 to −0.57) and a positive estimate in Erfurt (0.25; 95% CI, –0.81 to 1.31). There was evidence of the effect of PM_2.5_ from oil combustion only for SDNN among nonmedicated subjects. Crustal PM_2.5_ was associated with increased HF irrespective of medication use at lag 2. Associations between 5-day average (lags 0–4) particulate air pollution and HRV were weaker than for individual lags (data not shown).

The fraction of PM_2.5_ that could not be linked to any particular source category was positively associated at 0-day lag with SDNN (estimate 0.18; 95% CI, 0.00 to 0.35) and HF (1.53; CI, 0.48 to 2.59) among all study subjects, but the association was not evident among subjects not using beta-blockers. The positive association between unidentified PM_2.5_ fraction and SDNN disappeared when extreme source-specific PM_2.5_ concentrations were excluded from the analyses. Overall, exclusions of extreme values did not change the interpretation of the results. After exclusion, the city-specific estimates were no longer heterogeneous for the association of long-range transported PM_2.5_ with HF at lag 2 among all study centers.

Among persons not having daily beta-blocker medication, increases in absorbance (local traffic) and S (long-range transport) were consistently associated with decreased SDNN and HF (Table 5). The associations between V (oil combustion) and HRV were less consistent, and for the other source indicators there was no evidence of an effect. However, for the transition metals (Cu, Fe, and Zn) included because of potential toxicity, there was some evidence of negative associations with HRV at longer lags.

## Discussion

In this panel study conducted among persons with coronary heart disease in three European cities, personal, indoor, or outdoor PM_2.5_ measured during the 24 hr preceding clinic visit (lag 0) were not associated with HRV. However, at 2- and 3-day lags, we observed that daily increases in outdoor levels of PM_2.5_ were associated with decreased HRV, but only among persons not on beta-blocker medication. When we linked source-specific PM_2.5_to HRV, we observed increases in traffic-related PM_2.5_ to be associated with decreased SDNN, especially among persons who were not on beta-blocker medication. Daily increases in the long-range transported PM_2.5_ were associated both with decreased HF and SDNN, more strongly or exclusively among nonmedicated persons. In separate analyses, indicator elements for these two sources, absorbance and S, were also negatively associated with HRV among persons not on medication. There was also evidence for a negative association of transition metals with HRV.

We reported previously that outdoor levels of PM_2.5_ were not consistently associated with HRV in the three study panels (Timonen et al. 2006). However, people spend most of their time indoors, and persons with compromised health, like the panel members in our study, even more so (Brunekreef 2005). Consequently, outdoor levels of particulate air pollution measured at a central site may not be perfect proxies for variation in personal PM exposure. However, we did not find personal or indoor PM_2.5_ to be associated with decreased HRV. Unfortunately, we had only personal and indoor measurements in the 24 hr preceding the clinic visit, and PM_2.5_ mass and composition during that time period were not associated with HRV. Our observation thus indicates only that the lack of association at 0-day lag for outdoor PM_2.5_ was not due to exposure misclassification. In some studies, the effects of PM on HRV have been observed even within hours of exposure (Devlin et al. 2003; Gold et al. 2000). However, the use of daily averages to measure PM_2.5_ exposure in our study prevented us from detecting possible immediate effects of PM.

Beta-blockers have been shown to enhance HRV in patients with coronary heart disease (Niemela et al. 1994; Sandrone et al. 1994). Consistent with this, we observed increased outdoor levels of PM_2.5_ to be associated with decreased SDNN and HF (at 2- and 3-day lags) only among persons not using beta-blockers. Effect modification by medication use thus seems to explain the lack of associations between PM_2.5_ and HRV in our previous analysis (Timonen et al. 2006). There was little evidence of effect modification by any other medication group in the present study.

The interpretation of earlier studies evaluating the importance of beta-blocker use for the effects of ambient particles on HRV is somewhat difficult because of the differences in disease status between users and nonusers of beta-blockers. In a study by Park et al. (2005) conducted among veteran men, beta-blocker users were all hypertensive, whereas only half of the nonusers had hypertension. No clear effect of PM_2.5_ (adjusted for ozone) on SDNN or HF was observed in either medication group. However, the low-frequency component of HRV decreased in association with PM_2.5_ only among persons not using beta-blockers. In a study by Wheeler and coworkers (2006), all but one of the beta-blocker users were myocardial infarction survivors, whereas most nonusers had chronic obstructive pulmonary disease. Effect modification by beta-blocker use was reported only for SDNN, which decreased in association with PM_2.5_ among users and increased among nonusers. In the present study, all patients had coronary heart disease, and our results suggest that the use of beta-blockers modifies the effect of PM on HRV even in this more homogeneous patient group.

Medication use is obviously never independent of health status. Consequently, the suggestive increase in HF in association with PM_2.5_ among beta-blocker users in our study may indicate either that the use of medication changes the direction of the association, or that those with less severe heart disease differ in their response to particulate air pollution. Obesity has been suggested to modify the effects of PM on HRV (Chen et al. 2007), which was confirmed by our results. PM_2.5_ seemed to be more strongly associated with HRV among obese persons. In our study, obesity was not associated with beta-blocker use.

Clinical studies have related decreased HRV in cardiac patients with increased risk of mortality over relatively long periods of follow-up (Task Force 1996). The extent to which short-term decreases in HRV measures predict short-term mortality is not known. However, vagal withdrawal is observed a few minutes before transient ischemic events (Kochiadakis et al. 2000; Kop et al. 2001), suggesting that short-term changes in HRV are not harmless. In a large study among elderly subjects (de Bruyne et al. 1999), increased HRV has been even more strongly associated with decreased survival than decreased HRV. Taking this into account, our study cannot be straightforwardly interpreted as showing that beta-blocker use is protective against the effects of particulate air pollution on cardiovascular health, because there was a suggestive increase in HF in association with PM_2.5_ among medicated persons.

There was some indication of the effects of traffic-related PM_2.5_ on SDNN, and long-range transported PM_2.5_ on HF and SDNN even before taking medication into account, but after considering beta-blocker use, the associations became stronger. Some earlier studies have evaluated the effects of traffic-related particles on HRV without conducting source apportionment. Absorbance, considered as an indicator for traffic-originating particles, has been more strongly associated with HRV among elderly subjects than PM_2.5_ (Schwartz et al. 2005). In-vehicle PM_2.5_ was more strongly associated with HRV in healthy young men than were ambient or roadside PM_2.5_ (Riediker et al. 2004a). In-vehicle PM_2.5_ was further apportioned among different sources (Riediker et al. 2004b), and strongest associations were observed between PM_2.5_ from brake wear and engine emissions and HRV.

Schwartz et al. (2005) evaluated indirectly the effects of secondary particles on HRV and found no effect. They regressed PM_2.5_ against black carbon concentrations and interpreted residuals to represent the fraction of secondary particles that varied independently from primary combustion particles. It is possible that the effects of long-range transported PM_2.5_ on HRV in our study are related to primary combustion particles generated, for example, by regional traffic. In our study, the effect estimates (for SDNN) per microgram of particle mass were clearly higher for local traffic-related PM_2.5_ than for long-range transported particles. However, there was also some evidence of the effects of PM_2.5_ from oil combustion on SDNN. The results are consistent with our previous study, where PM_2.5_ from traffic and other local combustion was most strongly associated with the occurrence of ST segment depressions in Helsinki, but long-range transported particles and possibly oil combustion were also contributing to the effects of PM_2.5_ (Lanki et al. 2006).

In the last part of our analyses, we evaluated the associations of HRV with elements of PM_2.5_ and absorbance, a proxy for elemental carbon content of particles. In these analyses, decreased HRV was associated with absorbance and S, which were considered markers for local traffic and long-range transported PM_2.5_, respectively. The finding thus confirmed the analyses conducted using source-specific PM _2.5_. However, long-range transported PM_2.5_ also contains traffic-originating PM and elemental carbon. There was also evidence of the negative associations of V (oil combustion), Zn (e.g., industry), Fe, and Cu with HRV, but the associations were mostly nonsignificant. Transition metals are typically associated with combustion processes, so it was not a surprise that absorbance was highly correlated with Zn, Fe, and Cu. It has been suggested that organic carbon compounds and transition metals attached to elemental carbon core (approximated by absorbance) are responsible for the effects of PM on health (Obot et al. 2002).

Toxicologic studies have often observed cellular defenses to be even more responsive to the coarse particle fraction (PM_10_–PM_2.5_) than to finer-size fractions (Hetland et al. 2004; Soukup and Becker 2001). The ambient levels of coarse particles are typically dominated by crustal material, whereas PM_2.5_ levels are more influenced by combustion emissions. In a recent study (Lipsett et al. 2006), coarse particles were associated with decreased HRV, whereas PM_2.5_ was not. Interestingly, we found increases in HF in association with increased outdoor levels of crustal PM_2.5_. On the other and, the chosen indicator element for crustal PM_2.5_—Ca—was not associated with HF.

Our study has both strengths and weaknesses. The study had rather stringent inclusion criteria for the study subjects to obtain a homogeneous cardiac panel presumably vulnerable for the effects of air pollution (von Klot et al. 2005). The three study centers used common standard operating procedures and standardized equipment, and Holter recordings were analyzed in a single lab. HRV was recorded during a paced breathing period to avoid influence of breathing patterns on the results. However, because we measured out- door levels of source-specific PM _2.5_ instead of actual exposure, exposure misclassification may have biased the results. We previously reported considerable longitudinal correlations between outdoor and personal PM_2.5_, absorbance (traffic), and S (long-range transport), but correlations were lower for Ca (soil), Cl (salt), and Cu (Janssen et al. 2005). Finally, our source-specific PM_2.5_ levels are not always products of homogeneous sources but rather of broader source categories.

In conclusion, we found PM_2.5_ originating from local traffic and other local combustion and also long-range transported PM _2.5_ to be associated with decreased indices of HRV. The effects were stronger among persons not using beta-blocker medication and among obese persons. Differences in the composition of particles and medication use or disease severity of study subjects may explain some inconsistencies between previous studies on HRV.

## Figures and Tables

**Table 1:** Characteristics of three study panels.

Characteristic	Amsterdam (*n* = 33)^a^	Erfurt (*n* = 44)^a^	Helsinki (*n* = 45)^a^
Sex/female [no. (%)]	11 (34)	4 (9)	21 (47)
Mean age (range)	70.9 (54–83)	64.3 (40–78)	68.2 (54–83)
Obese^b^ [no. (%)]	7 (19)	7 (15)	16 (34)
Past myocardial infarction [no. (%)]	22 (69)	30 (68)	27 (60)
Angina pectoris [no. (%)]	21 (66)	24 (55)	29 (64)
CABG or PTCA conducted [no. (%)]	17 (53)	31 (70)	23 (51)
Daily beta-blocker medication [no. (%)]	13 (39)	34 (77)	31 (69)
Ca^2+^ channel blockers [no. (%)]	11 (30)	18 (38)	13 (28)
Statins [no. (%)]	12 (32)	20 (43)	21 (45)
ACE inhibitors and angiotensin receptor blockers [no. (%)]	12 (32)	25 (53)	10 (21)
ASA [no. (%)]	22 (59)	36 (77)	36 (77)
Mean^c^ SDNN (SD), msec			
Beta-blocker users	46.7 (17.8)	29.9 (8.8)	35.3 (10.7)
Nonusers	40.0 (11.9)	41.2 (12.1)	38.8 (11.7)
Mean^c^ HF (SD), msec^2^			
Beta-blocker users	414 (289)	280 (237)	600 (376)
Nonusers	504 (307)	547 (396)	629 (409)

Abbreviations: CABG, coronary artery bypass graft; PTCA, percutaneous transluminal coronary angioplasty.

a Number of patients available for analyses.

b Obese = body mass index ≥ 30 kg/m^2^.

c Average of individual means and SDs.

**Table 2:** Daily outdoor levels of PM_2.5_, its components, and temperature at central measurement sites in three cities.

	Amsterdam (*n* = 223)				Erfurt (*n* = 156)				Helsinki (*n* = 164)			
	p25	p50	P75	p95	p25	p50	p75	p95	p25	p50	p75	p95
PM_2.5_ (μg/m^3^)	10.4	16.7	23.9	47.0	10.8	16.3	26.7	62.3	8.3	10.6	15.9	25.8
Source-specific PM_2.5_ (μg/m^3^)												
Local traffic	3.5	6.1	9.3	20.4	4.1	7.0	10.0	18.4	1.7	2.6	3.4	6.5
Long-range transported	0.3	5.1	11.6	21.8	3.1	5.4	9.8	31.9	2.2	5.5	9.8	15.9
Oil combustion	0.9	1.6	3.1	5.9	NA	NA	NA	NA	0.6	1.3	2.3	4.2
Industry	−2.6	−0.5	3.0	9.2	−3.6	−1.6	2.2	24.7	NA	NA	NA	NA
Crustal	0.7	1.4	2.1	3.6	1.8	2.7	4.8	13.8	0.0	0.4	1.1	2.2
Salt	0.1	0.2	0.8	1.8	NA	NA	NA	NA	0.3	0.8	1.2	2.4
Absorbance (m^−1^ × 10^−5^)	0.9	1.5	2.2	3.4	1.3	2.0	3.4	5.1	1.4	1.89	2.47	3.56
Elements (ng/m^3^)												
S	936	1,340	2,240	3,650	600	862	1,530	3,740	839	1,380	2,080	3,400
V	2.5	4.1	7.8	14.7	NA	NA	NA	NA	3.2	6.69	9.8	16.4
Zn	8.7	18.2	33.9	65.2	22.6	40.2	75.1	199	11.3	16.8	25.1	47.3
Ca	26.9	37.1	51.8	76.9	34.6	47.0	72.2	193	22.7	32.3	47.3	87.0
Cl	33.8	116	432	990	NA	NA	NA	NA	8.1	36.4	102	386
Fe	47.1	70.7	107	175	38.6	59.9	112	248	39.3	66.7	100	165
Cu	1.4	2.5	4.7	9.0	0.6	2.5	4.9	10.4	0.6	1.6	2.8	5.1
Temperature (°C)	4.4	7.9	12.3	16.9	−0.1	3.9	6.7	11.2	−5.0	−0.6	2.5	8.8

Abbreviations: NA, not available; p25, 25th percentile; p50, 50th percentile (median); p75, 75th percentile; p95, 95th percentile.

**Table 3:** Correlation (Spearman’s correlation coefficients.) of total PM_2.5_ with source-specific PM_2.5_ and elements at central sites in three cities.

	Source-specific PM_2.5_						Elements of PM_2.5_							
	Traffic	LRT	Oil	Industry	Crustal	Salt	ABS	S	V	Zn	Ca	Cl	Fe	Cu
PM_2.5_
Amsterdam (*n* = 223)	0.50	0.62	0.18	0.27	−0.15	0.04	0.73	0.84	0.27	0.81	0.04	0.14	0.68	0.63
Erfurt (*n* = 156)	0.32	0.57	NA	0.41	0.19	NA	0.81	0.85	NA	0.82	0.51	0.63	0.81	0.70
Helsinki (*n* = 164)	0.26	0.82	0.35	NA	−0.01	0.19	0.70	0.85	0.59	0.77	0.17	−0.03	0.38	0.42

Abbreviations: NA, not available; LRT, long-range transported.

**Table 4:** Pooled effect estimates in three study panels [β (95% CIs)]^a^ for the associations of source-specific PM_2.5_ with HRV in multipollutant models.^b^

	SDNN (msec)		HF (%)	
	All subjects	Subjects without beta-blockers	All subjects	Subjects without beta-blockers
Local traffic				
Lag 0	−0.05 (−0.26 to 0.15)	0.11 (−0.23 to 0.44)	0.11 (−1.05 to 1.28)	0.31 (−1.65 to 2.30)
Lag 1	−0.12 (−0.36 to 0.12)	−0.27 (−0.59 to 0.05)	0.43 (−0.91 to 1.79)	−0.21 (−2.16 to 1.77)
Lag 2	−0.28 (−0.57 to 0.01)	−0.45 (−0.90 to 0.01)	−0.13 (−1.74 to 1.50)	−0.67 (−3.34 to 2.07)
Lag 3	−0.20 (−0.45 to 0.06)	−0.35 (−0.69 to 0.00)	−0.64 (−2.03 to 0.78)	−1.43 (−3.40 to 0.58)
Long-range transport				
Lag 0	0.00 (−0.10 to 0.09)	−0.03 (−0.19 to 0.14)	0.12 (−0.43 to 0.67)	−0.18^c^ (−1.13 to 0.77)
Lag 1	−0.04 (−0.14 to 0.06)	0.00 (−0.15 to 0.16)	0.19 (−0.38 to 0.77)	0.06 (−0.86 to 0.99)
Lag 2	−0.05 (−0.17 to 0.07)	−0.11 (−0.30 to 0.07)	−0.69^c^ (−1.35 to −0.02)	−1.06 (−2.14 to 0.03)
Lag 3	0.00 (−0.13 to 0.12)	−0.20 (−0.39 to −0.01)	−0.54 (−1.23 to 0.15)	−1.98 (−3.07 to −0.88)
Oil combustion^d^				
Lag 0	−0.02 (−0.74 to 0.70)	−0.46 (−1.34 to 0.41)	3.20 (−0.48 to 7.03)	1.43 (−3.83 to 6.97)
Lag 1	−0.29 (−1.04 to 0.45)	−1.08 (−2.09 to −0.06)	1.05 (−2.70 to 4.94)	−3.04 (−8.80 to 3.08)
Lag 2	0.36 (−0.42 to 1.13)	0.22 (−0.89 to 1.33)	1.50 (−2.36 to 5.51)	0.10 (−6.34 to 6.98)
Lag 3	0.00 (−0.77 to 0.77)	−0.43 (−1.27 to 0.42)	0.49 (−3.25 to 4.38)	−0.42 (−5.32 to 4.73)
Industry^d^				
Lag 0	−0.07 (−0.23 to 0.09)	−0.17 (−0.43 to 0.10)	0.13 (−0.80 to 1.07)	0.08 (−1.44 to 1.62)
Lag 1	0.03 (−0.12 to 0.19)	−0.14 (−0.44 to 0.16)	0.62 (−0.34 to 1.59)	−0.08 (−1.79 to 1.65)
Lag 2	0.02 (−0.12 to 0.16)	−0.08 (−0.34 to 0.18)	0.05 (−0.82 to 0.94)	−1.03 (−2.53 to 0.49)
Lag 3	−0.04 (−0.17 to 0.09)	0.12 (−0.19 to 0.42)	−0.05 (−0.87 to 0.77)	0.68 (−0.98 to 2.37)
Crustal				
Lag 0	−0.02 (−0.36 to 0.31)	−0.05 (−0.84 to 0.75)	0.01 (−2.07 to 2.15)	0.80 (−3.47 to 5.26)
Lag 1	0.11 (−0.35 to 0.56)	0.07 (−0.97 to 1.11)	1.57 (−1.28 to 4.50)	1.93 (−3.86 to 8.06)
Lag 2	0.18 (−0.37 to 0.73)	0.35 (−0.82 to 1.52)	4.72 (1.16 to 8.41)	5.67 (−1.11 to 12.91)
Lag 3	0.11 (−0.43 to 0.66)	0.20 (−1.05 to 1.45)	0.93 (−2.43 to 4.41)	2.68 (−4.02 to 9.84)
Salt^d^				
Lag 0	1.07 (−0.66 to 2.80)	−0.03 (−2.61 to 2.55)	5.20 (−3.83 to 15.08)	4.33 (−10.46 to 21.56)
Lag 1	−0.19 (−1.92 to 1.55)	−0.64 (−3.29 to 2.00)	−1.43 (−9.86 to 7.78)	4.33 (−10.68 to 21.87)
Lag 2	−0.33 (−2.13 to 1.47)	−0.44 (−2.88 to 2.00)	−1.06 (−9.69 to 8.38)	−6.55 (−18.85 to 7.62)
Lag 3	1.47 (−0.28 to 3.22)	2.17 (−0.07 to 4.41)	6.70 (−2.30 to 16.52)	2.74 (−9.65 to 16.83)

a β, effect estimate for an increase of 1 μg/m^−3^ in source-specific PM_2.5_.

b The number of observations in the analyses was 1,195 for SDNN and 1,183 for HF.

c Pooled effect estimates have heterogeneous underlying center-specific effect estimates (significance test < 0.05).

d Oil combustion source and salt source of PM_2.5_ were not identified in Erfurt; industrial source of PM_2.5_ was not identified in Helsinki; estimates of only two cities pooled.

**Table 5:** Pooled effect estimates [β (95% CIs)]^a^ in three study panels for the associations of elements of PM with HRV among study subjects in single-pollutant models.

	SDNN (msec)		HF (%)	
	All subjects	Subjects without beta-blockers	All subjects	Subjects without beta-blockers
ABS				
Lag 0	−0.54 (−1.39 to 0.31)	−0.64 (−2.25 to 0.97)	0.45 (−4.48 to 5.64)	−2.54 (−11.15 to 6.91)
Lag 1	−0.52 (−1.46 to 0.41)	−1.59 (−3.11 to −0.06)	2.91 (−2.54 to 8.67)	−4.94 (−13.04 to 3.91)
Lag 2	−0.78 (−1.72 to 0.16)	−1.36 (−2.99 to 0.27)	−1.42 (−6.76 to 4.22)	−7.13 (−15.51 to 2.08)
Lag 3	−0.31 (−1.23 to 0.62)	−1.44 (−3.15 to 0.27)	−2.57 (−7.75 to 2.90)	−7.83 (−16.27 to 1.45)
S				
Lag 0	−0.25 (−1.06 to 0.55)	−0.71 (−1.98 to 0.56)	0.74 (−3.76 to 5.46)	−2.70 (−9.71 to 4.84)
Lag 1	−0.51 (−1.36 to 0.33)	−0.76 (−1.99 to 0.47)	0.25^b^ (−4.42 to 5.14)	−3.61 (−10.36 to 3.64)
Lag 2	−0.43 (−1.39 to 0.52)	−1.44 (−2.84 to −0.04)	−4.78^b^ (−9.69 to 0.40)	−10.56 (−17.63 to −2.87)
Lag 3	0.10 (−0.88 to 1.08)	−1.54 (−3.02 to −0.06)	−4.02 (−9.01 to 1.25)	−13.05 (−20.18 to −5.29)
V^c^				
Lag 0	−0.12 (−1.14 to 0.91)	−0.46 (−1.85 to 0.93)	4.58 (−0.89 to 10.36)	3.68 (−4.72 to 12.82)
Lag 1	−0.66 (−1.73 to 0.41)	−1.97 (−3.56 to −0.39)	0.73 (−4.74 to 6.53)	−6.24 (−14.71 to 3.07)
Lag 2	0.40 (−0.66 to 1.46)	−0.16^b^ (−1.72 to 1.39)	1.40 (−4.02 to 7.13)	−4.58 (−12.97 to 4.61)
Lag 3	0.04 (−0.99 to 1.07)	−0.43 (−1.78 to 0.91)	−1.92 (−7.00 to 3.45)	−2.09 (−9.64 to 6.09)
Zn				
Lag 0	−0.19 (−0.79 to 0.41)	−0.69 (−1.83 to 0.46)	1.68 (−1.97 to 5.46)	0.40 (−5.91 to 7.13)
Lag 1	0.12 (−0.55 to 0.79)	−0.78 (−2.11 to 0.54)	3.85 (−0.26 to 8.13)	0.13 (−6.92 to 7.72)
Lag 2	0.06 (−0.58 to 0.70)	−0.92 (−2.20 to 0.37)	2.28 (−1.71 to 6.43)	−3.78 (−10.41 to 3.35)
Lag 3	−0.13 (−0.72 to 0.46)	−0.28 (−1.53 to 0.96)	−1.43 (−4.95 to 2.22)	−6.41 (−12.60 to 0.22)
Ca				
Lag 0	−0.23 (−0.85 to 0.38)	−0.72 (−2.17 to 0.73)	−0.77 (−4.50 to 3.10)	−0.37 (−8.12 to 8.04)
Lag 1	0.27 (−0.58 to 1.11)	−0.47 (−2.16 to 1,21)	3.39 (−1.80 to 8.86)	2.10 (−7.18 to 12.31)
Lag 2	0.62 (−0.36 to 1.60)	0.35 (−1.47 to 2.18)	7.89 (1.70 to 14.46)	5.60 (−4.98 to 17.35)
Lag 3	0.03 (−0.93 to 1.00)	0.01 (−2.03 to 2.05)	0.61 (−5.01 to 6.56)	−0.01 (−10.58 to 11.81)
Cl^c^				
Lag 0	0.25 (−0.25 to 0.76)	0.07 (−0.48 to 0.61)	2.36 (−0.15 to 4.94)	2.34 (−1.00 to 5.79)
Lag 1	0,14 (−0.39 to 0.67)	0.00 (−0.63 to 0.64)	1.13 (−1.48 to 3.81)	1.46 (−2.39 to 5.46)
Lag 2	0.38 (−0.12 to 0.88)	0.32 (−0.22 to 0.85)	1.40 (−1.06 to 3.94)	1.71 (−1.56 to 5.08)
Lag 3	0.31 (−0.17 to 0.80)	0.37 (−0.13 to 0.87)	1.21 (−1.15 to 3.61)	0.81 (−2.21 to 3.91)
Fe				
Lag 0	−0.32 (−1.25 to 0.61)	−0.32 (−1.94 to 1.29)	0.72 (−4.49 to 6.22)	−0.12 (−9.19 to 9.86)
Lag 1	0.15 (−1.00 to 1.30)	−1.09 (−2.85 to 0.67)	6.69 (0.11 to 13.69)	0.13 (−9.70 to 11.04)
Lag 2	−0.44 (−1.72 to 0.84)	−1.51 (−3.58 to 0.56)	1.50 (−5.52 to 9.04)	−3.31 (−14.26 to 9.05)
Lag 3	−0.44 (−1.67 to 0.79)	−1.77 (−3.86 to 0.32)	−3.45 (−9.90 to 3.46)	−9.93 (−20.26 to 1.72)
Cu				
Lag 0	−0.18 (−0.73 to 0.36)	−0.29 (−1.26 to 0.68)	1.56 (−1.65 to 4.87)	−0.97 (−6.40 to 4.77)
Lag 1	−0.08 (−0.74 to 0.57)	−0.20 (−1.19 to 0.78)	3.00 (−0.85 to 7.00)	2.34 (−3.49 to 8.52)
Lag 2	−0.43 (−1.10 to 0.24)	−1.55 (−2.71 to −0.39)	1.71 (−2.30 to 5.88)	−4.16 (−10.53 to 2.67)
Lag 3	0.12 (−0.56 to 0.80)	−0.54 (−1.59 to 0.51)	−1.97 (−5.76 to 1.98)	−4.41 (−9.91 to 1.43)

a β, effect estimate, calculated for an increase of 1 m^−1^ × 10^−5^ in absorbance, 1 μg/m^3^ in S, 4 ng/m^3^ in V, 30 ng/m^3^ in Ca and Zn, 100 ng/m^3^ in Cl, 70 ng/m^3^ in Fe, and 2 ng/m^3^ in Cu.

b Pooled effect estimates have heterogeneous underlying center-specific effect estimates (significance test < 0.05).

c V and Cl were not used in Erfurt; estimates of only two cities pooled.
